# The Discovery of Insulin-Regulated Aminopeptidase (IRAP) Inhibitors: A Literature Review

**DOI:** 10.3389/fphar.2020.585838

**Published:** 2020-09-23

**Authors:** Dimitris Georgiadis, Angeliki Ziotopoulou, Eleni Kaloumenou, Angelos Lelis, Antonia Papasava

**Affiliations:** Laboratory of Organic Chemistry, Department of Chemistry, National and Kapodistrian University of Athens, Athens, Greece

**Keywords:** inhibitor, insulin-regulated aminopeptidase, inhibitor selectivity, aminopeptidase, structure-activity relationships

## Abstract

Insulin-Regulated Aminopeptidase (IRAP, EC 3.4.11.3) is a multi-tasking member of the M1 family of zinc aminopeptidases. Among its diverse biological functions, IRAP is a regulator of oxytocin levels during late stages of pregnancy, it affects cellular glucose uptake by trafficking of the glucose transporter type 4 and it mediates antigen cross-presentation by dendritic cells. Accumulating evidence show that pharmacological inhibition of IRAP may hold promise as a valid approach for the treatment of several pathological states such as memory disorders, neurodegenerative diseases, etc. Aiming to the investigation of physiological roles of IRAP and therapeutic potential of its regulation, intense research efforts have been dedicated to the discovery of small-molecule inhibitors. Moreover, reliable structure-activity relationships have been largely facilitated by recent crystal structures of IRAP and detailed computational studies. This review aims to summarize efforts of medicinal chemists toward the design and development of IRAP inhibitors, with special emphasis to factors affecting inhibitor selectivity.

## Introduction

Insulin-regulated aminopeptidase (IRAP, oxytocinase, EC 3.4.11.3) is a type II transmembrane Zn-protease that belongs to the M1 family of aminopeptidases (Rogi et al., 1996; Laustsen et al., 1997; Nomura et al., 2013). IRAP was so named because it was first identified in specialized vesicles in fat and muscle cells co-localized with the glucose transporter GLUT4. Upon insulin receptor stimulation these vesicles translocate to plasma membrane to facilitate glucose uptake into the cells (Keller et al., 1995). IRAP is also called oxytocinase since it was also isolated from the placenta and was found to regulate the levels of circulating oxytocin during the later stages of human pregnancy (Rogi et al., 1996). IRAP is highly expressed in brain regions associated with cognition (Fernando et al., 2005) and is able to degrade macrocyclic peptides, such as oxytocin and vasopressin, which are known to influence favorably cognitive functions (Gulpinar and Yegen, 2004; Wallis et al., 2007; Rimmele et al., 2009; Albiston et al., 2011). Moreover, IRAP is inhibited by angiotensin IV (AngIV), hence IRAP was recognized as a potential target for the treatment of cognitive disorders during the last decade (Albiston et al., 2001; Lew et al., 2003; Albiston et al., 2008; Albiston et al., 2011; Andersson and Hallberg, 2012). Except from IRAP’s association to glucose metabolism and cognition-related functions, it is also involved in the generation of antigenic MHC peptides for cross-presentation (Saveanu et al., 2009; Saveanu and Van Endert, 2012), in the trafficking of T-cell receptors (Evnouchidou et al., 2020) and in the progress of cardiac and renal fibrosis (Gaspari et al., 2018a; Gaspari et al., 2018b). Although the development of IRAP inhibitors was initially aiming to the development of cognitive enhancers, applications in the regulation of immune responses or as antifibrotic agents are also emerging fields of pharmacological interest. In this mini-review, we present the different classes of IRAP inhibitors, we briefly discuss key interactions that govern inhibitor binding and we provide selectivity profile data and possible interpretation of observed selectivity. We must note that *K*_i_ or IC_50_ values of inhibitors mentioned in this review may have been estimated using different types of assays. Regardless of the methodology, general conclusions on the comparison of inhibition profiles are not significantly affected by this discrepancy.

## Types of IRAP Inhibitors

### AngIV Peptidic Analogs and Peptidomimetics

Undoubtedly, a strong inspiration toward the development of the first IRAP inhibitors came from the breakthrough discovery that IRAP is identified as the AT_4_ receptor (Albiston et al., 2001). Based on prior work of Harding and Wright on AngIV analogs with high affinity for AT_4_ (Sardinia et al., 1993; Sardinia et al., 1994; Krishnan et al., 1999), Lew et al. demonstrated that AT_4_ receptor ligands, such as Nle^1^–AngIV, divalinal-AngIV, decapeptide LVVYPWTQRF (LVV-hemorphin-7) and the parent peptide AngIV, inhibit the proteolytic activity of IRAP in HEK293T cell membranes with *K*_i_ values between 113 nM and 2.3 μM (Lew et al., 2003). In 2003, Lee et al. produced several truncated or alanine-substituted analogs of LVV-hemorphin-7 and evaluated their binding affinity in competition studies with ^125^I-AngIV in sheep adrenal and cerebellar membranes (Lee et al., 2003). The authors also estimated the *K*_i_ values in an enzyme inhibition assay for selected truncated LVV-hemorphin-7 analogs which revealed that *N*-terminal amino acid deletion can be tolerated up to Val^3^ whereas *C*-terminal deletion does not significantly affect binding affinity up to Pro^6^. Interestingly, tripeptide VYP is only three time less potent inhibitor (*K*_i_ = 620 nM) than the parent decapeptide (*K*_i_ = 196 nM), highlighting the importance of hydrophobic residues for binding affinity against IRAP.

**Figure 1:**
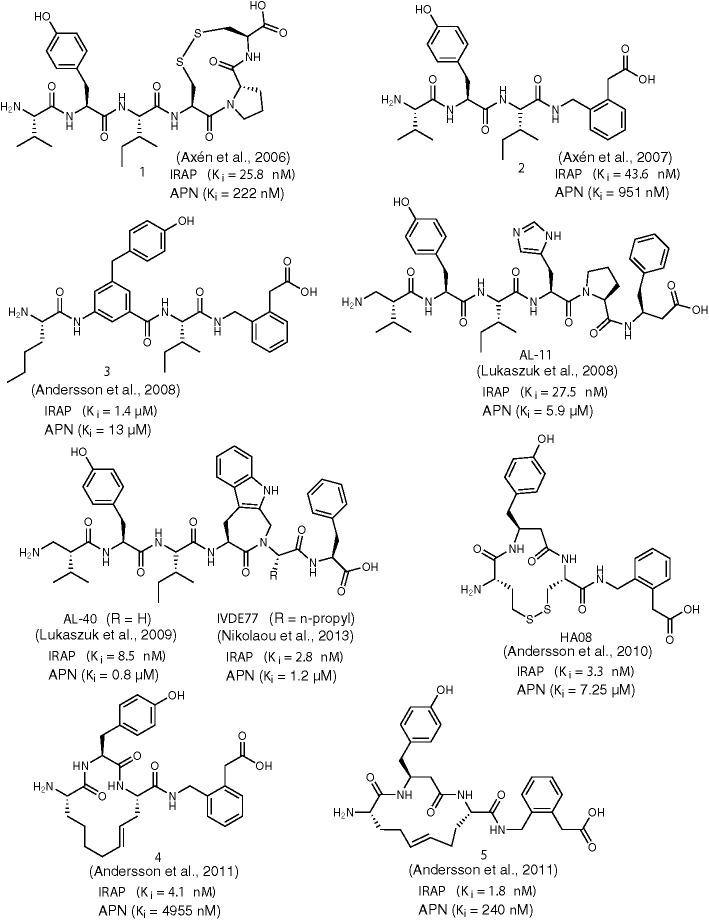
Chemical structures of AngIV-based inhibitors of IRAP **1**-**5**, **AL-11**, **AL-40**, **IVDE77,** and **HA08**. *K*_i_ values for IRAP and APN are given in all cases.

In 2006, the research group of Hallberg designed and evaluated a series of disulfide-cyclized AngIV analogs based on the assumption that macrocyclization would confer metabolic stability toward enzymatic degradation while retaining the high affinity of AngIV for IRAP (Axén et al., 2006). Indeed, compound 1 (**Figure 1**) was identified which encompasses an 11-membered, conformationally flexible ring, displays similar inhibitory potency toward IRAP as AngIV and is characterized by reduced susceptibility against proteolysis. The significance of proper positioning of disulfide linkage within the peptide became evident after detailed evaluation of several suitably designed AngIV analogs, which revealed that only cyclization at the *C*-terminal region of AngIV can lead to inhibitors equipotent to AngIV. This observation led to the conclusion that the *Cys* ^4^-Pro^5^-*Cys* ^6^ moiety in compound 1 adopts an inverse *γ*-turn conformation that possibly resembles the bioactive conformation of AngIV, as it was further supported by conformational analysis. These findings triggered subsequent studies by the same group aiming to the gradual transition from AngIV peptide analogs to more “drug-like”, designed peptidomimetics. In a 2007 report, it was proposed that the conformational characteristics of the *C*-terminal sequence His^4^-Pro^5^-Phe^6^ of AngIV (or the *Cys* ^4^-Pro^5^-*Cys* ^6^ tail of compound 1) can be mimicked by a non-peptidic 2-(aminomethyl)phenylacetic acid (AMPAA) moiety which optimally positions the terminal carboxylate for efficient enzyme recognition (Axén et al., 2007). Compound 2 (**Figure 1**) showed considerably higher metabolic stability against metalloproteases present in CHO-K1 cell membranes than AngIV and it was able to induce proliferation of adult neural stem cells. Moreover, compound 2 was almost twice more selective for IRAP versus APN than the disulfide analog 2, which validates furthermore Hallberg’s rigidification approach.

In 2008, Hallberg and co-workers extended their previous studies on backbone modification of AngIV by introducing additional non-cleavable amino acid surrogates, in order to increase metabolic stability of IRAP inhibitors (Andersson et al., 2008). Among the various peptidomimetics developed, compound 3 (**Figure 1**) showed the highest potency for IRAP (*K*_i_ = 1.4 μM), albeit 23-fold lower than AngIV. As compared to AngIV structure, Tyr^2^ was replaced by a 4-hydroxydiphenylmethane scaffold and *C*-terminal sequence His^4^-Pro^5^-Phe^6^ was replaced by an AMPAA moiety. A conceptually similar approach was employed by Lukaszuk et al. who produced analogs of AngIV with *β* ^2^- or *β* ^3^-amino acid substitutions in order to enhance potency, stability and IRAP/APN selectivity (Lukaszuk et al., 2008). Compound AL-11 (**Figure 1**) was particularly interesting to that respect since it was able to inhibit IRAP with a *K*_i_ value of 27.5 nM, it displayed a ~200-fold selectivity for APN and it was completely stable in the presence of CHO-K1 cell membranes. The development of AL-11 was based on the observation that the introduction of *β* ^2^-hVal^1^ and *β* ^3^-hPhe^6^ substitutions contribute to the metabolic stability and the suppression of binding to AT1, respectively, whereas the synergy of both substitutions is responsible for the reported selectivity for IRAP versus APN. The same research team managed to improve inhibitory potency for IRAP by replacing His^4^-Pro^5^ of AngIV by Aia^4^-Gly^5^ (Aia: 4-amino-1,2,4,5-tetrahydro-indolo[2,3-c]-azepin-3-one) while maintaining the *β* ^2^-hVal^1^ substitution (AL-40, **Figure 1**) (Lukaszuk et al., 2009). Evidently, the introduction of a constrained Trp analog instead of His^4^ is responsible for a substantial conformational transformation of AL-40 which boosts binding affinity for IRAP. Similar replacements of Tyr^2^ by constrained amino acids had a deleterious effect on potency whereas such modifications for Pro^4^ and Phe^6^ are well tolerated, albeit without surpassing the efficiency of AL-40 (Lukaszuk et al., 2011). Finally, screening of *α*-substituted amino acids in lieu of Gly^5^ of AL-40 led to the discovery of inhibitor IVDE77 (**Figure 1**) that shows remarkable efficiency in terms of potency for IRAP (*K*_i_ = 1.7 nM), IRAP/APN and IRAP/AT1 selectivity and metabolic resistance against proteolytic degradation (Nikolaou et al., 2013). Moreover, by using a tritiated analog of the inhibitor, it was demonstrated that IVDE77 was able to completely eliminate IRAP availability at the cell surface *in vitro*.

Concurrently with the work of Belgian researchers that culminated in the discovery of IVDE77, Hallberg and co-workers refined the structure of previously reported AngIV-based inhibitors bearing a *C*-terminal AMPAA motif (e.g. compound 2) by imposing conformational constraints at the *N*-terminal region (Andersson et al., 2010). Prior work had shown that replacement of Val^1^ and Ile^3^ of AngIV with Cys residues followed by oxidative cyclization causes a dramatic drop in IRAP inhibitory activity (*K*_i_ = 16.9 μM), suggesting that the *N*-terminal of AngIV is very sensitive to conformational changes (Axén et al., 2006). However, potency was significantly restored (*K*_i_ = 303 nM) when hCys instead of Cys residues were employed, thus expanding the initial 11-membered to a 13-membered ring. Interestingly, replacement of the *C*-terminal His^4^-Pro^5^-Phe^6^ with the AMPAA motif, not only resulted to a 13-fold increase in IRAP inhibitory potency but also abolished activity toward APN. Further structural modifications led to optimized inhibitor HA08 (**Figure 1**) which displays excellent IRAP potency and IRAP/APN selectivity and encompasses a *β* ^3^-hTyr^2^-Cys^3^ sequence, thus retaining the privileged 13-membered ring size. Very recently, Mpakali et al. solved the crystal structure of IRAP/HA08 complex and identified several key features of the inhibition mechanism (Mpakali et al., 2020). The authors propose that upon binding, HA08 induces an extensive conformational change to IRAP which corresponds to the so-called “closed” state, previously observed also in the case of an IRAP inhibitor of phosphinic type (*vide infra*). This conformational transition establishes tight interactions between the macrocyclic inhibitor and the GAMEN loop of IRAP, a common structural feature of M1 aminopeptidases which in the case of IRAP presents unusual conformational plasticity and unprecedented adaptability. Despite HA08’s peptidic nature, its tolerance to degradation by IRAP relies on the tight spatial juxtaposition with the GAMEN loop that precludes water molecules to be activated by Glu465 and trigger catalysis.

Unfortunately, unlike IRAP, other proteases are able to degrade HA08, rendering this inhibitor metabolically unstable. To this regard, Andersson et al. developed a series of improved macrocyclic analogs by applying a ring-olefin metathesis protocol, in order to replace the labile disulfide bond of HA08 with a carbon-carbon bond (Andersson et al., 2011). Gratifyingly, researchers succeeded in producing proteolytically stable derivatives showing excellent potency for IRAP and IRAP/APN selectivity, as it is exemplified in the most efficient inhibitors of this series, compounds 4 and 5 (**Figure 1**). In contrast to HA08, optimized inhibitors 4 and 5 encompass an one-carbon expanded 14-membered ring, a structural feature that improves binding affinity by one order of magnitude, implying that the size of the macrocycle critically affects the conformational characteristics of the inhibitor. Efforts to bridge positions 1 and 3 of AngIV macrocyclic peptidomimetics by an amide bond had a negative effect in inhibitory potency, as it was described in a recent report (Barlow et al., 2020). According to molecular dynamics analysis, this behavior could be explained by an unfavorable orientation of the AMPAA moiety inside the active-site pocket, due to conformational changes induced by the presence of amide linkage.

### 4*H*-Benzopyrans

**Figure 2:**
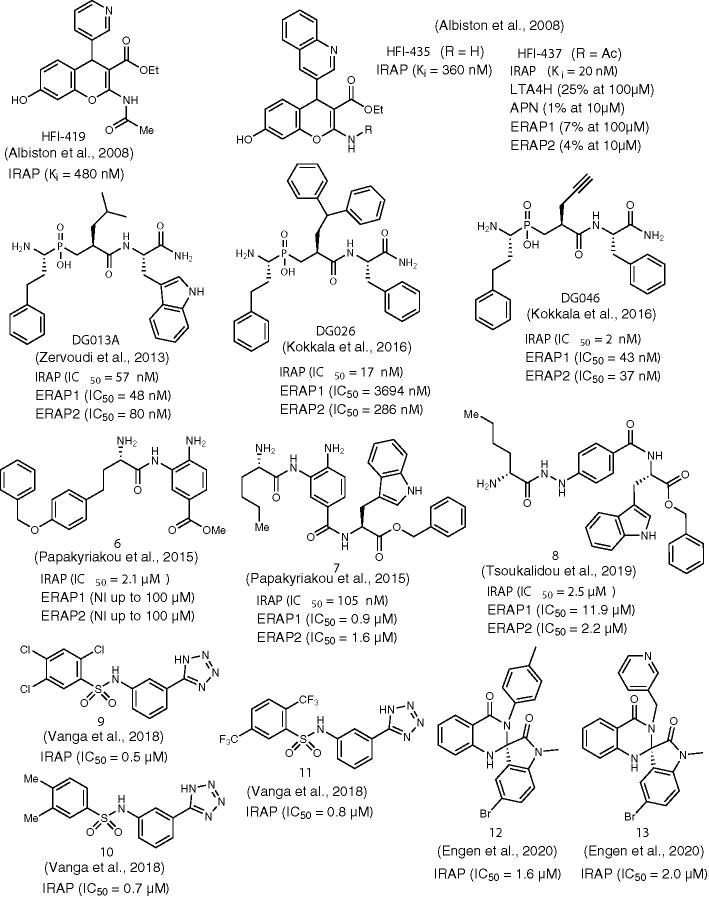
Chemical structures of non-AngIV based inhibitors of IRAP **6-13**, **HFI-410**, **HFI-435**, **DG013A**, **DG026**, and **DG046**. *K*_i_ or IC_50_ values for IRAP are given in all cases. *K*_i_ or IC_50_ values for other aminopeptidases are also provided, if available.

Another milestone discovery in the history of IRAP inhibitors appeared in the literature in 2008 by a group of Australian researchers (Albiston et al., 2008). By performing virtual screening of a database of 1.5 million compounds against an homology model of IRAP, the team identified the 2-amino-4*H*-benzopyran scaffold as a promising hit for further development. Indeed, after evaluating several benzopyran analogs and applying medicinal chemistry modifications to the most active lead, compounds HFI-419, HFI-435, and HFI-437 (**Figure 2**) were selected as those exhibiting *K*_i_ values lower than 1 μM. These were the first IRAP inhibitors that were not based on AngIV or other peptide scaffolds. All of the inhibitors were selective for IRAP versus other aminopeptidases such as LTA4H, APN, ERAP1, and ERAP2, as it is exemplified in the case HFI-437 in **Figure 2**. All compounds were tested as racemates, however the *S*-isomer is believed to be more active, as it was suggested by molecular docking that revealed a better fit to the catalytic site of IRAP (based on a LTA4H crystal structure) (Albiston et al., 2010). During these initial docking efforts, two different poses were predicted as optimal for pyridine and quinoline derivatives. In both cases, interactions with Phe544 appeared to be crucial for binding, as it was further verified by site-directed mutagenesis experiments. However, revisited predictions after the resolution of empty IRAP’s crystal structure showed both scaffolds dock with almost identical poses, with the hydroxyl group on the benzopyran ring interacting with Zn^2+^ and the benzopyran ring juxtaposing against the GAMEN loop (Hermans et al., 2015). This binding pose accurately explains SAR data showing the complete loss of activity after replacement of the hydroxyl group at 7-position of 4*H*-benzopyrans that was initially erroneously attributed to disruption of critical hydrogen bonding interactions with active site residues (Mountford et al., 2014). Moreover, according to revisited predictions, the aromatic system of pyridine or quinoline interacts with Phe544 to a different degree, which could explain the greater sensitivity of quinoline derivatives to mutations at residue Phe544. HFI-419 exhibited better aqueous solubility and allowed the improvement of spatial working and recognition memory in rodents, possibly by increasing hippocampal dendritic spine density *via* a GLUT4-mediated mechanism (Seyer et al., 2019).

### Phosphinic Pseudopeptides

Among its several biological functions, IRAP has been implicated in an intracellular pathway that generates antigenic peptides for cross-presentation by dendritic cells (Saveanu et al., 2009; Saveanu and Van Endert, 2012). IRAP is highly homologous to ERAP1 and ERAP2, two other intracellular M1 aminopeptidases that are also key players in the trimming of antigenic peptide precursors to optimal length for loading onto MHC class I molecules. In 2003, in the quest of ERAPs inhibitors that could potentially regulate immune responses, with possible applications ranging from cancer immunotherapy to treatment of inflammatory autoimmune diseases, phosphinic pseudotripeptide DG013A (**Figure 2**) was developed being a potent inhibitor of all three enzymes, with a *K*_i_ value for IRAP of 57 nM (Zervoudi et al., 2013). The mechanism of inhibition by this rationally designed inhibitor was further investigated by solving the crystal structure of ERAP2/DG013A complex which revealed that the hydroxyphosphinyl moiety interacts with Zn^2+^ through the two oxygen atoms of tetrahedral phosphorous, forming also hydrogen bonds with Glu371 and Tyr455, two residues critical for catalysis. Based on the analogy of DG013A binding pose with the conformation of a substrate during the transition state of hydrolytic reaction, a typical property of phosphinic pseudopeptide protease inhibitors (Georgiadis and Dive, 2015), a subsequent SAR study was performed aiming to unveil those structural determinants that confer selectivity between ERAPs and IRAP (Kokkala et al., 2016). Two inhibitors derived from this study displayed distinct properties in terms of IRAP inhibition: DG026 and DG046 (**Figure 2**).

DG026, which includes a bulky benzydryl group in its P_1_΄ position, showed high potency and selectivity for IRAP vs ERAPs (115-fold for ERAP1 and 23-fold for ERAP2) and succeeds in reducing IRAP-dependent but not ERAP1-dependent cross-presentation by dendritic cells with nanomolar efficacy. Aiming at understanding the reasons behind this inhibition profile, Mpakali et al. solved the crystal structure of IRAP/DG026 complex which revealed for the first time a “closed” conformational state for IRAP that is believed to be induced upon ligand binding (Mpakali et al., 2017b). This conformation was known for ERAPs but it had never been observed for IRAP. Indeed, both IRAP structures available at that time, one ligand-free and one bound with an antigenic epitope-based phosphinic pseudopeptide, were found in an open or semi-closed conformation, respectively (Hermans et al., 2015; Mpakali et al., 2015). In the transition from empty IRAP to the closed conformation of IRAP/DG026, a large rearrangement of GAMEN loop takes place [as it was later observed in the IRAP/HA08 crystal structure (Mpakali et al., 2020)] that is not common in other aminopeptidases and may account for the wider substrate specificity of IRAP. The selectivity of DG026 could probably be attributed to unfavorable repulsive interactions of bulky hydrophobic benzhydryl goup with the overall more polar environment of ERAPs, compared to IRAP. Furthermore, it was proposed based on structural analysis that the high potency and selectivity of DG026 is a direct result of its near-optimal complementarity for IRAP, creating a network of interactions that probably drive the conformational change from the open to the closed state.

Interestingly, DG046 bearing a small, linear propargylic side chain at P_1_΄ position is one of the most potent IRAP inhibitors ever reported (*K*_i_ = 2 nM) with a fairly good selectivity versus ERAP1 and ERAP2 by a factor of ~20 for both. Probably, the proposed plasticity of IRAP’s active site leads to potent inhibitors with either small or bulky groups at P_1_΄ position. Recently, the ERAP1/DG046 structure became available which provides important insights on the selectivity determinants of DG046 (Giastas et al., 2019). In particular, stacking interactions of Tyr residues in the case of ERAP2 and IRAP with the phenyl ring of P_2_΄ position of DG046 cannot take place in ERAP1 that has a Ser at the same position. Finally, the side chain of Ile461 in the case of IRAP could properly orientate propargyl group toward a zinc coordinating histidine and favor the formation of critical π–π interactions.

### 3,4-Diaminobenzoic Acid Derivatives

Another class of compounds that have been extensively evaluated as inhibitors of IRAP and ERAPs by Vourloumis group are 3,4-diaminobenzoic acid (DABA) derivatives bearing natural and/or unnatural aminoacids (Papakyriakou et al., 2013; Papakyriakou et al., 2015). Selectivity for IRAP was feasible in the expense of potency, as it is exemplified in the case of compound 6 (**Figure 2**), an IRAP inhibitor of IC_50_ = 2.1μM which was inactive against ERAPs up to 100μM (Papakyriakou et al., 2015). Improvement of binding affinity led to reduced selectivity, as depicted in **Figure 2** for compound 7. In fact, compound 7 (IC_50_ = 105nM) was the most potent inhibitor of all 77 compounds tested in this study for all three enzymes, which emphasizes the overall moderate efficacy of DABA-based inhibitors. Indeed, structural analysis of a DABA derivative with ERAP2 revealed a rather weak interaction between the carbonyl oxygen of DABA core with the zinc ion (Mpakali et al., 2017a). Further, variations of the same structural theme led to only moderate, non-selective IRAP inhibitors with compound 8 being the most representative example (Tsoukalidou et al., 2019).

### Aryl Sulfonamides

In 2014, Swedish researchers recognized the ability of aryl sulfonamides to inhibit IRAP after screening a library of 10,500 drug-like compounds (Borhade et al., 2014; Engen et al., 2016). After a hit-to-lead process, several compounds of moderate potency were selected, such as 9 and 10 which were shown to alter dendritic spine morphology and increase spine density in primary cultures of hippocampal neurons (Diwakarla et al., 2016). Recently, further lead optimization furnished several fluorinated analogs (e.g. compound 11) that presented higher metabolic stability by human or mouse liver microsomes (Vanga et al., 2018). In the absence of any IRAP structure, initial attempts to rationalize inhibitor binding and interpret SAR data by molecular docking concluded that the tetrazole ring may be involved in stabilizing interactions with Zn^2+^ and Ty549 (Borhade et al., 2014). However, guided by the IRAP/DG026 structure published in 2015 by Stratikos group (Mpakali et al., 2017b), subsequent extensive computational analysis qualified an alternative binding pose where it is an oxygen atom of sulfonamide that coordinates with Zn^2+^. Moreover, the amide NH is stabilized by interactions with the Glu residue of the GAMEN loop and tetrazole ring develops polar interactions with Arg439.

### Spiro-oxindole Dihydroquinazolinones

During the screening process that led to the discovery of aryl sulfonamide inhibitors, one of the most potent hits was compound 12 (**Figure 2**) based on the spiro-oxindole dihydroquinazolinone scaffold (Engen et al., 2016). Efforts to improve the poor solubility of hit compound 12 (e.g. compound 13, **Figure 2**) led in all cases to derivatives with reduced metabolic stability *in vitro* (Engen et al., 2020). Nevertheless, this class of compounds presents high specificity for IRAP vs APN. According to docking calculations, a unique feature of spiro-oxindole derivatives is that the inhibitor in its preferred binding pose is positioned in close proximity with the GAMEN loop without interacting with Zn^2+^, leading to uncompetitive inhibitors as it was further supported by kinetic experiments.

## Conclusions

After two decades of intense efforts toward the discovery of IRAP inhibitors, it would not be an exaggeration to say that the field is still in its infancy. Recent crystal structures of IRAP with bound small-molecule inhibitors are expected to rationalize binding preferences of known inhibitors and facilitate the discovery of new ones, either by accelerating the hit-to-lead optimization process during screening endeavours or by guiding efforts toward structure-based rational design or scaffold hopping. Of course, a question that needs to be addressed is whether the “closed” conformational state of the enzyme and the GAMEN loop re-orientation observed in recent structures of IRAP with DG026 and HA08 (Mpakali et al., 2017b; Mpakali et al., 2020), constitute a prerequisite for efficient binding of small-molecule inhibitors or not. Recently, Vanga et al. predicted structural determinants for IRAP inhibition by aryl sulfonamides using IRAP’s “open” structure, however attempts to re-examine their predictions by using the “closed” structure of IRAP were unsuccessful due to the inability of inhibitors to access the catalytic site of the enzyme (Vanga et al., 2018). The authors formulate the assumption that in this specific case stabilization of an “open” enzyme conformation would probably be more favourable. Similarly, Mpakali et al. observed substantial differences between preferred binding modes of benzopyran inhibitor HFI-437 when docked to the empty IRAP “open” structure, as compared to the IRAP/DG025 “closed” one (Mpakali et al., 2017b). Taking into account the unique, ligand-induced active-site plasticity of IRAP, possible identification of additional conformational states of IRAP induced by inhibitors of other types will complicate even more the already perplexed mechanism of IRAP inhibition. Inspiration for the design of novel inhibitors can be drawn by scaffolds that have been successfully evaluated against other M1 aminopeptidases but not for IRAP (e.g. boronic acids, aminothiols, bestatin derivatives etc) (Mucha et al., 2010). However, extreme caution is required when such inhibitors are evaluated in order to avoid possible cross-reactivity which may trigger off-target side-effects (Drinkwater et al., 2017). Undoubtedly, delineation of binding particularities associated with different inhibitor scaffolds is expected to reveal those specific interactions that enhance inhibitor selectivity and eventually lead to next-generation inhibitors with safer inhibition profiles and improved pharmacological properties.

## Author’s Note

Dedicated to the memory of our dear colleague Dr. Dionisios Vourloumis.

## Author Contributions

EK, AZ, AP, and AL conducted the review of literature, analyzed and organized bibliography, prepared the figures and drafted part of the manuscript. DG conceived the project, drafted part of the manuscript, revised and finalized the manuscript. All authors contributed to the article and approved the submitted version.

## Funding

This work was supported by funds from the Special Account for Research Grants of NKUA.

## Conflict of Interest

The authors declare that the research was conducted in the absence of any commercial or financial relationships that could be construed as a potential conflict of interest.
